# Clostridium septicum Gas Gangrene Complicated by Cerebral Air Embolism

**DOI:** 10.7759/cureus.85614

**Published:** 2025-06-09

**Authors:** Tânia Lopes, Paulo Conceição, António Costa, Rita Rego, Ana Rita Cruz

**Affiliations:** 1 Department of Internal Medicine, Centro Hospitalar Universitário de Santo António, Porto, PRT; 2 Department of Neurology, Centro Hospitalar Universitário de Santo António, Porto, PRT

**Keywords:** cerebral air embolism, clostridium septicum, colorectal cancer, gas gangrene, necrotizing fasciitis, pneumocephalus, soft tissue infection

## Abstract

*Clostridium septicum* (*C. septicum*) is a rare cause of necrotizing soft tissue infection and is most commonly associated with spontaneous infections in patients with underlying gastrointestinal pathology. In rare cases, it can affect other organs or cause air embolism. We report the case of an 85-year-old man with diabetes mellitus who initially presented with fever and later developed inflammatory signs in the left upper limb and chest wall. CT revealed signs of gas gangrene in these locations. Due to the onset of prostration and motor aphasia, a brain CT scan was performed and showed an area of infarction in the left anterior cerebral artery territory with gas bubbles inside, likely secondary to air embolism. Empirical antibiotic therapy was initiated, and the patient underwent surgical debridement. *C. septicum* was isolated from both blood cultures and debrided tissue samples. Despite an initially satisfactory clinical response, the patient ultimately passed away on the seventeenth day of hospitalization. In necrotizing soft tissue infections, rapid diagnosis and treatment are critically important due to their high morbidity and mortality rates. In survivors of *C. septicum* infection, colorectal cancer should always be ruled out.

## Introduction

Necrotizing soft tissue infection is a rare clinical condition, usually caused by a loss of skin integrity, that progresses rapidly and can be fatal if not treated promptly [1]. The majority of these infections are polymicrobial, caused by a combination of aerobic and anaerobic bacteria [1]. Monomicrobial necrotizing infections include gas gangrene caused by Clostridium species, which are uncommon pathogens in these types of infections [1]. Clostridium perfringens is the most frequently identified species and is typically associated with trauma or postoperative complications [1].

*Clostridium septicum* (*C. septicum*) is rarely identified in necrotizing soft tissue infections and is associated with spontaneous infections in the vast majority of cases [2]. Most patients have an underlying GI pathology, with colorectal cancer being the most prominent [3, 4]. Other risk factors for *C. septicum* infection include hematologic malignancies, diabetes mellitus (DM), neutropenia, and immunosuppression [2, 3, 5]. *C. septicum* infection is associated with high morbidity and mortality rates, reaching 70-80% in some studies, with most deaths occurring within the first 48 hours due to septic shock [2, 3, 5, 6].

Spontaneous *C. septicum* gas gangrene has an initially insidious clinical course, presenting with pain in the affected area. The classical signs, including inflammatory changes, bullae, and crepitus, typically appear later, followed by rapid clinical deterioration [2, 5]. Besides gas gangrene, *C. septicum* infection can have several other presentations, as it may affect various organs such as the liver, aorta, brain, eye, heart, and large joints [2, 5]. Additionally, sepsis or bacteremia may develop [2, 5].

Hematogenous spread to the CNS is a rare complication of *C. septicum* bacteremia and may present as meningitis, encephalitis, or brain abscess [3, 4, 7]. These conditions can be associated with pneumocephalus, since *C. septicum* is capable of producing gas, although this is a rare complication [3, 4, 7]. Another potential consequence of gas production is air embolism, though only rare cases have been described in the literature [8, 9]. We present a unique case of *C. septicum* gas gangrene with cerebral air embolism to add to the existing literature, as this is an extremely rare complication of the disease.

## Case presentation

The patient was an 85-year-old Caucasian man with insulin-dependent type 2 DM, diagnosed seven years earlier, with good metabolic control but complicated by diabetic retinopathy and nephropathy. His medical history also included hypertension, dyslipidemia, and chronic iron deficiency anemia. Two months prior, he had sustained a left trochanteric fracture and underwent surgical correction without complications. The patient was admitted to the emergency department with a one-day history of fever, without any focal symptoms. On admission, his Glasgow Coma Scale (GCS) score was 15; he was oriented to time and place, febrile, and hemodynamically stable, with no significant physical findings, including neurologic examination.

Blood tests revealed severe anemia, thrombocytopenia, Kidney Disease: Improving Global Outcomes (KDIGO) stage 1 acute kidney injury (AKI), and a mild elevation in C-reactive protein (CRP) (Table 1). Arterial blood gas analysis was unremarkable. Chest radiography showed no lung infiltrates or consolidations. Urine sediment analysis revealed no signs of infection. Abdominopelvic ultrasound showed no abnormalities. The patient received one unit of RBC transfusion and remained under observation for etiologic investigation of the fever and abnormal laboratory findings.

**Table 1:** Evolution of the patient’s blood test results over time. D0: On admission; D1: 24 hours after admission; D2: 48 hours after admission.

Parameter (unit)	D0	D1	D2	Normal Range
Leukocytes (×10³/µL)	8.52	10.37	5.82	4-11
Neutrophils (×10³/µL)	7.34	8.88	4.66	2-7.5
Hemoglobin (g/dL)	7	8.2	8.5	13-17
Platelets (×10³/µL)	131	98	70	150-400
Creatinine (mg/dL)	1.56	1.39	2.27	0.7-1.2
Urea (mg/dL)	87	89	115	10-50
Myoglobin (µg/L)	122	516	7031	28-72
Creatine kinase (U/L)	75	359	3020	24-204
C-reactive protein (mg/L)	15	309	428	0-5

Approximately 24 hours after admission, the patient developed respiratory, cardiovascular, and neurological dysfunctions, presenting with a somnolent state and a GCS score of 10 (O3V2M5), without apparent focal neurological signs. Blood tests revealed neutrophilia, rising CRP levels, and rhabdomyolysis (Table 1). Empirical antibiotic therapy with piperacillin/tazobactam was promptly initiated, along with fluid resuscitation and oxygen therapy.

After approximately 48 hours of admission, the patient experienced persistent fever, further elevation in CRP, and worsening rhabdomyolysis, AKI, and thrombocytopenia (Table 1). At this point, his GCS score had improved slightly to 12 (O4V2M6), but motor aphasia was noted, without other focal neurological deficits. Inflammatory signs were identified in the left upper limb and lateral chest wall, along with a bulla in the axillary region, though without evidence of skin integrity loss.

**Figure 1:**
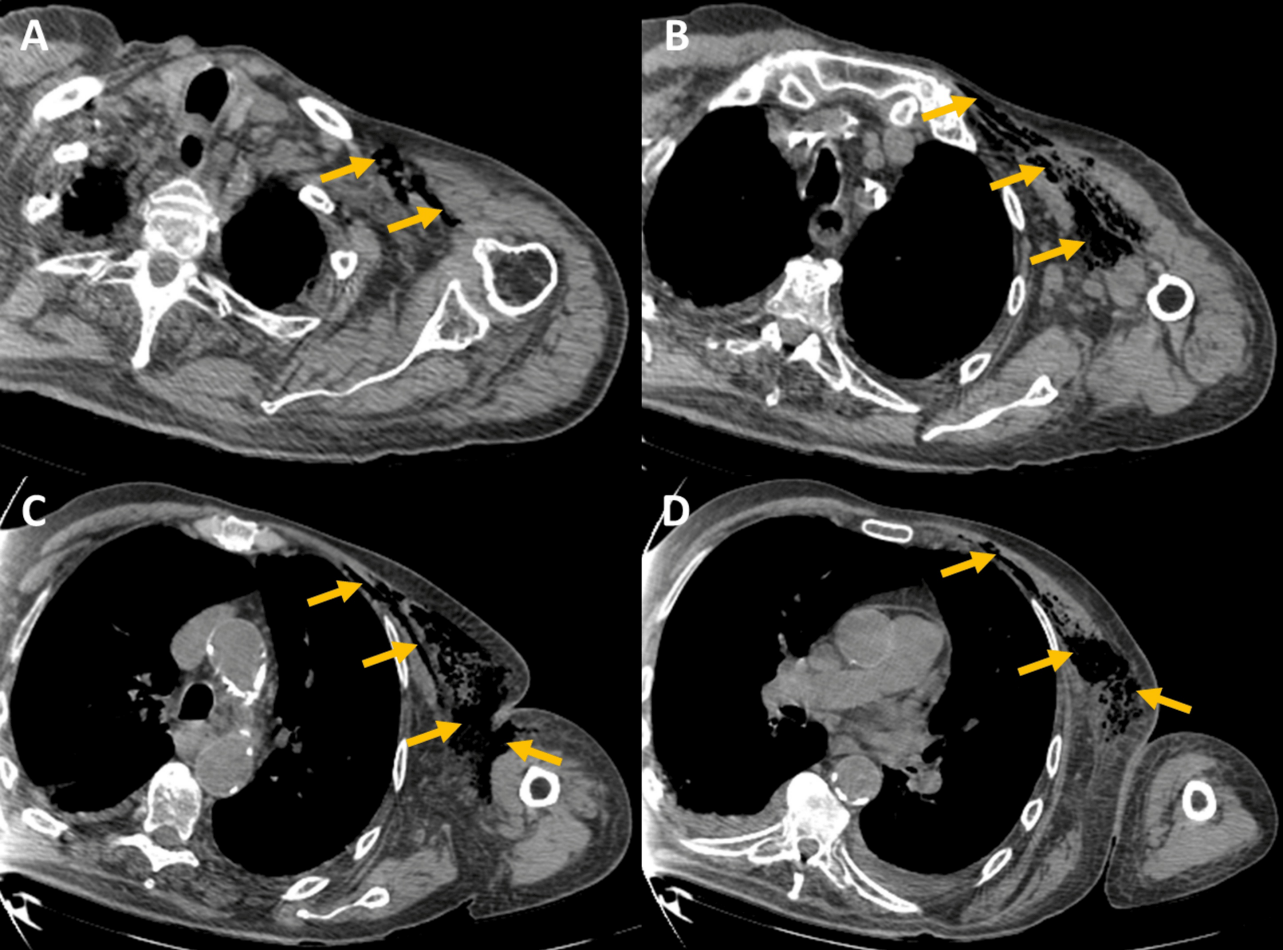
Chest and left upper limb CT scan, shown in multiple axial planes (A-D), revealing gas collections within the soft tissues (yellow arrows) and extensive densification of the subcutaneous fat in the left anterior chest wall and left upper limb.

CT of the chest and left upper limb (Figure 1) revealed gas collections within the soft tissues and extensive densification of the subcutaneous fat in the left anterior chest wall and left upper limb, findings highly suggestive of a necrotizing infection. No evidence of pneumothorax was seen. Empirical antibiotic therapy was adjusted to ceftriaxone, metronidazole, and daptomycin, and the patient underwent urgent surgical drainage and debridement of the affected area.

**Figure 2:**
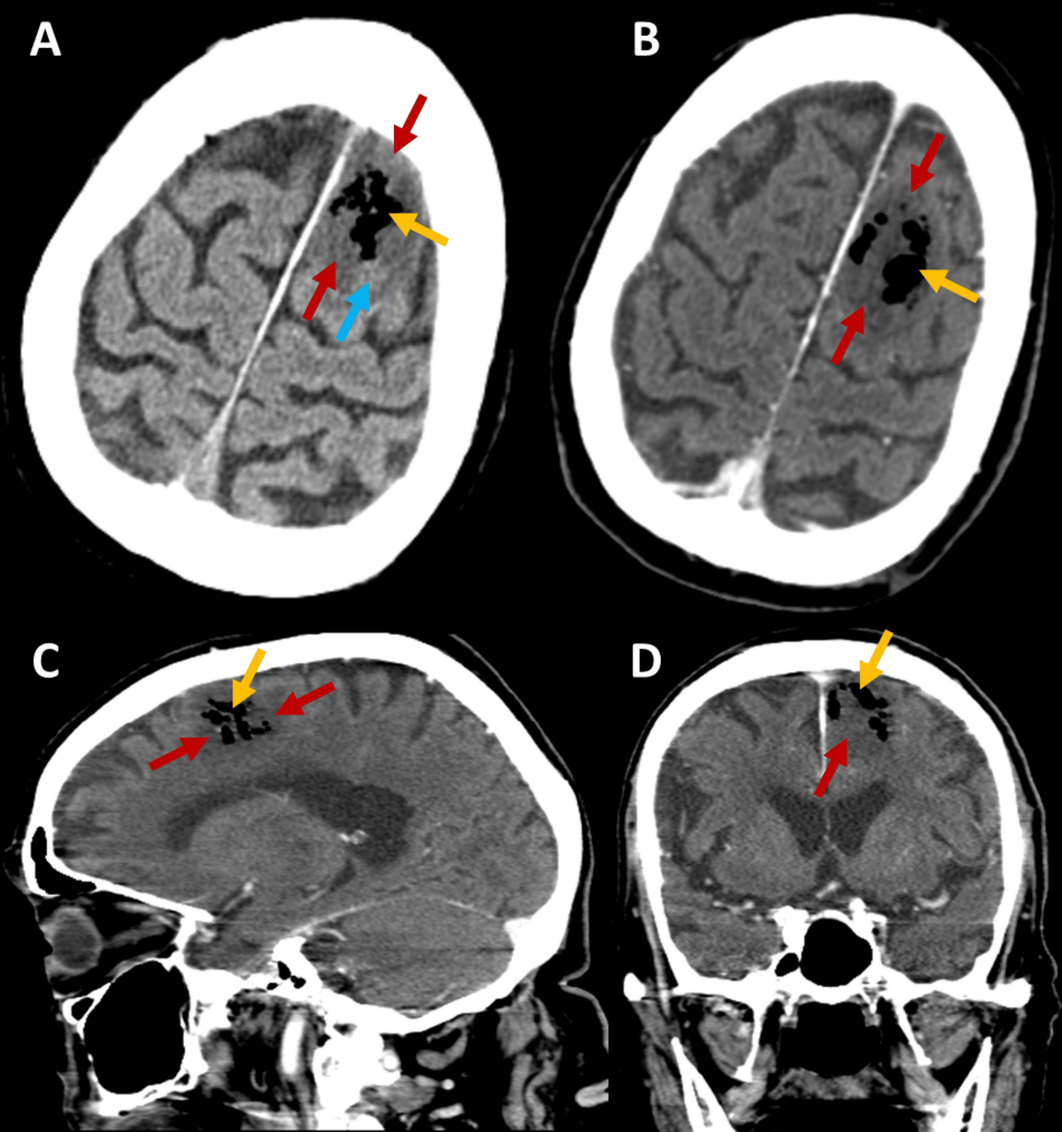
Brain CT scan images with contrast (B-D) and without contrast (A), revealing a cortico-subcortical hypodense lesion in the left frontal lobe (red arrows) with gaseous content (yellow arrows), shown in axial (A-B), sagittal (C), and coronal (D) planes. The blue arrow indicates an area of higher spontaneous density within the lesion.

Brain CT scan (Figure 2) showed a parasagittal cortico-subcortical hypodense lesion in the left frontal lobe with gaseous content and some small areas of higher spontaneous density within the lesion. There was effacement of adjacent sulci but no mass effect on the ventricular system or midline shift, and no significant enhancement after contrast administration. The lesion was compatible with a vascular insult in the territory of a distal branch of the left anterior cerebral artery (ACA), likely caused by air embolism, and was accompanied by pneumocephalus and areas suggestive of subacute intraparenchymal hemorrhage. The absence of significant enhancement after contrast administration made a brain abscess less likely, although encephalitis could not be ruled out. MRI was performed for further clarification.

**Figure 3:**
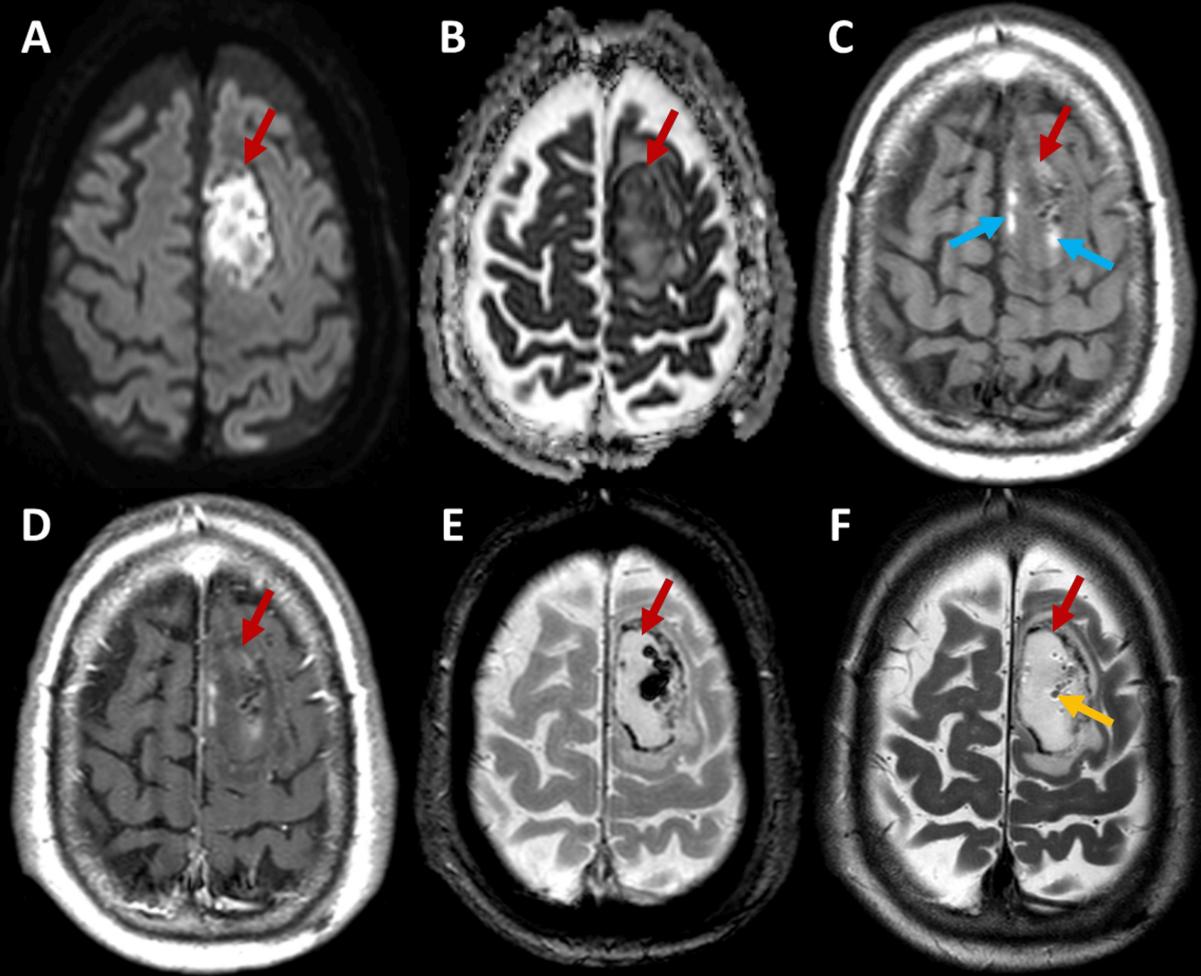
Brain MRI images in axial planes (A-F) demonstrate an ovoid lesion in the left frontal lobe (red arrows) with a gaseous component (yellow arrow). The lesion shows significant diffusion restriction and peripheral regions suggestive of a hemorrhagic component (blue arrows). No anomalous contrast uptake is observed.

Brain MRI (Figure 3), performed on the fifth day after admission, revealed a parasagittal ovoid lesion in the left frontal lobe with significant diffusion restriction, evidenced by hyperintensity on diffusion-weighted imaging (DWI) (Figure 3A) and corresponding hypointensity on the apparent diffusion coefficient (ADC) map (Figure 3B). Peripheral regions showed increased signal intensity on T1-weighted imaging (Figures 3C-3D) and decreased signal intensity on the T2-gradient echo-based sequence (Figure 3E), suggesting a late subacute hemorrhagic component. Additionally, rounded foci of low signal intensity within the lesion, better visualized on T2-weighted imaging (Figure 3F), were indicative of a gaseous component. Cortical veins and venous sinuses showed preserved flow. The lesion was associated with mild perilesional vasogenic edema, and there was no abnormal contrast uptake (Figure 3D). Minor ischemic areas in the same topography, distinct from the main lesion and containing small gas pockets, were also identified, features not previously reported on the initial CT scan.

Despite the lesion’s behavior on DWI, the presence of hemorrhagic components and the absence of abnormal contrast uptake supported the diagnosis of a vascular lesion in the context of air embolism, most likely arterial, and made infection less likely. Lumbar puncture was not performed due to the risk of cerebral herniation, as the presence of gas in the brain lesion could expand and exacerbate any mass effect. Therefore, although CNS infection could not be completely excluded, it was considered an unlikely diagnosis based on imaging findings, and there was no indication for neurosurgical intervention, as no areas suggestive of abscess were identified. A transthoracic echocardiogram showed no evidence of vegetations, and the bubble test excluded a right-to-left shunt.

**Figure 4:**
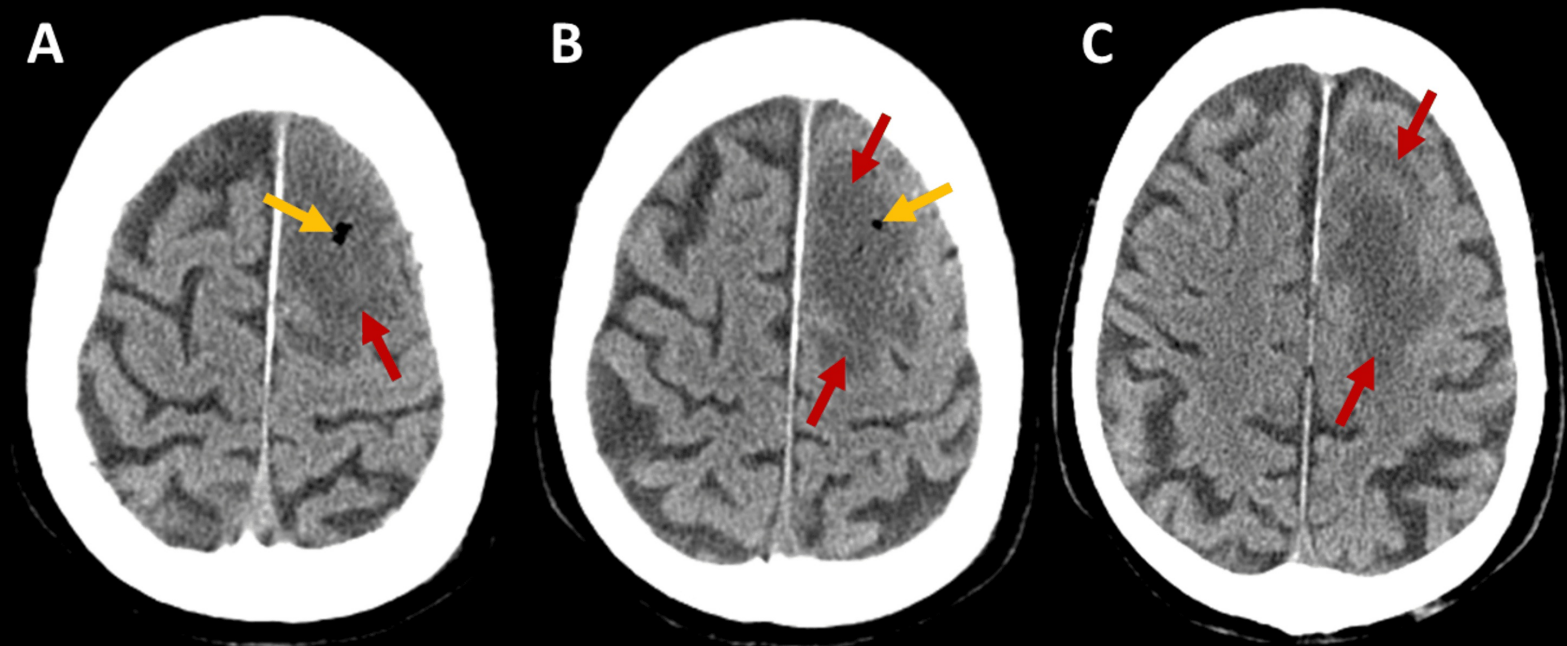
Brain CT scan in multiple axial planes (A-C) revealing an increase in the size of the hypodense lesion in the left frontal lobe (red arrows) with a reduction in the gaseous content (yellow arrows).

*C. septicum *was isolated from both blood cultures and debrided tissue samples. The patient initially showed favorable clinical evolution, with gradual improvement in organ dysfunction and resolution of all neurological deficits. A follow-up brain CT scan (Figure 4), performed on the ninth day after admission, showed an increase in the size of the known left frontal hypodense lesion and signs of gas bubble reabsorption. There was no significant mass effect, and the hyperdense components previously noted were no longer visible, most likely reflecting normal progression toward chronicity of the hemorrhagic content seen on MRI.

The patient unexpectedly passed away on the seventeenth day after admission, without any prior clinical deterioration that could have predicted such an outcome. In agreement with the family, no autopsy was performed.

## Discussion

*C. septicum* is a Gram-positive, anaerobic, spore-forming, and toxin-producing rod [2, 4, 6]. Among its toxins, alpha toxin is particularly noteworthy, as it is responsible for tissue necrosis and is the most lethal [4, 6, 7]. Studies estimate that this microorganism is present in the intestinal flora of only 2.8% of people, although some authors argue that it is part of the normal colonic flora [4]. It is known, however, to preferentially colonize the cecum and ileum [4, 6, 7]. Gastrointestinal pathology, such as neoplastic lesions, enterocolitis, radiation exposure, and cytotoxic or immunosuppressive drugs, can compromise the integrity of the gastrointestinal mucosa, leading to tissue hypoxia and facilitating the proliferation of *C. septicum* [4, 6]. This process leads to further mucosal ulceration, primarily through the action of alpha toxin, enabling vascular invasion and subsequent entry into the bloodstream [4, 6].

In a literature review by Macha K et al. that included patients with *C. septicum* CNS infection, 63% of those older than 65 years had colorectal cancer [3]. Therefore, survivors of *C. septicum* infections should always be screened for colorectal cancer due to the strong association between the two conditions [3]. It is important to note that our patient had chronic iron deficiency anemia that had not been investigated. Considering his age, a possible colorectal cancer could have been the entry site for *C. septicum*. However, endoscopic studies could not be performed during hospitalization to confirm this hypothesis. Additionally, DM has been associated with *C. septicum* gas gangrene in 41% of cases [2]. DM is well known to induce immunosuppression and predispose patients to gastrointestinal mucosal ulceration, facilitating hematogenous bacterial dissemination [2]. Therefore, it may have been a contributing factor to the infection in our patient. No direct link to the hip surgery was identified.

To date, no published cases have described cerebral air embolism in the context of *C. septicum* infection. However, there has been a case of gas gangrene caused by *C. septicum* with extensive gas embolism into the systemic circulation and multiple organs [8]. Additionally, another case of gas gangrene caused by Clostridium species reported peripheral arterial embolization, documented by Doppler ultrasound, in a patient with lower limb infection [9]. Thus, it is reasonable to conclude that gas-forming infections may cause air embolism in various locations, including the brain. Our patient presented with altered mental status and acute focal neurological deficits, both manifestations compatible with cerebral air embolism [10]. CT and MRI findings were also consistent with the hypothesis of air embolism as the cause of brain infarction and pneumocephalus.

Cerebral air embolism can originate from either the arterial or venous circulation [10, 11]. Arterial air embolism can occur through several mechanisms: direct entry of air into the systemic arterial circulation or pulmonary veins; entry of air into the venous system in volumes that exceed the filtration capacity of the pulmonary capillaries; or through the presence of a cardiac (e.g., patent foramen ovale or atrial septal defect) or pulmonary (e.g., arteriovenous malformation) right-to-left shunt [11, 12]. Venous air embolism results from retrograde progression of air through the jugular veins in an upright patient [11, 12].

Air would most likely enter the vascular system through the venous system, as it is the lower-pressure circuit [13]. In our patient’s case, venous air entry could have occurred in two potential ways: first, by diffusion of gas from extravascular tissues due to vascular permeability caused by infection-related endothelial damage [9]; second, iatrogenically, from venipuncture in the affected limb. However, the gas gangrene was limited to the proximal third of the arm, an uncommon site for peripheral venous catheter placement. As neither mechanism is likely to result in significant venous air entry, air would only reach the systemic circulation in the presence of a shunt, which was excluded by the bubble test. Therefore, both hypotheses appear unlikely.

Another consequence of air entry into the venous system, especially considering the location of the necrotizing infection, could have been retrograde venous air embolism. However, the imaging findings showed an ischemic lesion in a typical arterial territory, and the cortical veins and venous sinuses demonstrated preserved flow on MRI, suggesting arterial air embolism as the more likely cause; therefore, this hypothesis seems improbable. According to the authors, another potential and more plausible explanation for the presence of air in the vascular system would be gas production by circulating bacteria, either through gas formation within the systemic circulation, resulting in cerebral embolization, or, less likely, through gas production in the distal left ACA by lodged bacteria leading to infarction.

Although arterial cerebral air embolism can cause pneumocephalus, it remains a rare event [14]. Another possible explanation for the presence of pneumocephalus would be CNS infection. In a 2016 literature review by Macha K et al., 19 cases of CNS infection caused by *C. septicum* were identified [3]. Helmink AJ et al. conducted another review in 2023 and found two additional cases [4]. Of these 21 cases, 13 described the presence of pneumocephalus [4]. The case reported in their article was also complicated by pneumocephalus [4]. The mechanism by which the microorganism penetrates the blood-brain barrier is not fully understood, but endothelial injury caused by sepsis and microvascular destruction resulting from alpha toxin may contribute to intracerebral hemorrhage and facilitate dissemination of the microorganism into the brain parenchyma [4].

In fact, in the study by Helmink AJ et al., of the 21 reported CNS infection cases, six exhibited intraparenchymal hemorrhage, including the one described in their own report [4]. It is important to note that in our patient, imaging confirmed an area of intraparenchymal hemorrhage, which may have resulted from vascular fragility secondary to air embolism. Thus, if CNS infection was present, it appears to have been a secondary event following the air embolism, rather than the primary cause of the pneumocephalus. However, we cannot confirm or exclude the hypothesis of CNS infection, as no autopsy or lumbar puncture was performed, and imaging studies could not definitively rule out the possibility.

## Conclusions

This case highlights the importance of rapid diagnosis and treatment of necrotizing soft tissue infections due to their associated morbidity and mortality. However, in cases of *C. septicum* gas gangrene, prompt intervention can be challenging because of a relatively benign initial presentation, followed by rapid progression to sepsis and death. Therefore, maintaining a high index of suspicion is crucial. It is also important to recognize that organs beyond soft tissue can be affected, although these are less frequent manifestations of the disease. CNS is an uncommon but possible site of involvement. Extremely rare cases of air embolism have been reported in patients with Clostridium species infections. In this article, we describe a unique case of cerebral air embolism in a patient with *C. septicum* gas gangrene. In survivors of *C. septicum* infection, colorectal neoplasia should always be ruled out.
